# A signature based on NKG2D ligands to predict the recurrence of hepatocellular carcinoma after radical resection

**DOI:** 10.1002/cam4.5318

**Published:** 2022-10-09

**Authors:** Dongbo Chen, Jie Gao, Liying Ren, Pu Chen, Yao Yang, Shaoping She, Yong Xie, Weijia Liao, Hongsong Chen

**Affiliations:** ^1^ Peking University People's Hospital, Peking University Hepatology Institute Beijing Key Laboratory of Hepatitis C and Immunotherapy for liver Disease Beijing China; ^2^ Department of Hepatobiliary Surgery Peking University People's Hospital Beijing China; ^3^ Laboratory of Hepatobiliary and Pancreatic Surgery Affiliated Hospital of Guilin Medical University Guilin Guangxi China; ^4^ Division of Life Science The Hong Kong University of Science and Technology Hong Kong China; ^5^ Da Ren Biotech Limited Hong Kong China

**Keywords:** hepatocellular carcinoma, NKG2D ligands, recurrence, signature, ULBP3

## Abstract

**Introduction:**

Due to the high recurrence, the HCC prognosis remains poor. Yet, the biomarkers for predicting the recurrence of high‐risk patients are currently lacking. We aimed to develop a signature to predict the recurrence of HCC based on NKG2D ligands.

**Methods:**

The multivariate Cox proportional hazards regression was used to select recurrence‐related variables of NKG2D ligands in HCC patients from The Cancer Genome Atlas (TCGA). HCC patients from the OEP000321 dataset and Guilin cohort were used to validate the predictive signature. The mRNA expression of NKG2D ligands was measured by QRT‐PCR. Immunohistochemistry analysis of HCC tissue microarray samples was used to identify the expression of NKG2D ligands.

**Results:**

In this study, NKG2D ligands expression in the mRNA and protein level was both abnormally expressed in HCC and associated with recurrence‐free survival (RFS). Then, the recurrence‐related variables of NKG2D ligands in HCC were selected by the multivariate Cox proportional hazards regression. Among the eight NKG2D ligands, MICA (HR = 1.347; 95% CI = 1.012–1.793; *p* = 0.041), ULBP3 (HR = 0.453; 95% CI = 0.231–0.889; *p* = 0.021) and ULBP5 (HR = 3.617; 95% CI = 1.819–7.194; *p* < 0.001) were significantly correlated with RFS in the TCGA‐LIHC cohort. Then, the signature was constructed by the three NKG2D ligands. The predictive effectiveness of this signature was also validated in the OEP000321 dataset and Guilin cohort. Further, HCC patients were classified into low‐risk and high‐risk subgroups by the predictive score. Compared with the low‐risk group, the high‐risk group had poor RFS in both training and validation cohorts. Importantly, compared with the low‐risk patients with the G1‐G2 stage, the levels of infiltrated NK‐activated cells and NKG2D expression were both lower in the high‐risk patients.

**Conclusions:**

The signature based on MICA, ULBP3, and ULBP5 could predict HCC recurrence.

## Graphical abstract

NKG2D ligands expression in the mRNA and protein level was both abnormally expressed in HCC and associated with recurrence‐free survival (RFS). The signature based on MICA, ULBP3, and ULBP5 could predict HCC recurrence in three independent cohorts, which was related to the levels of NK‐cell infiltration in HCC tumor tissues. Our findings might provide a benefit in the management of postoperative patients of HCC.

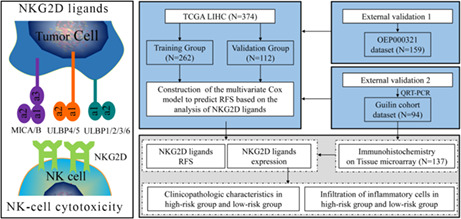

## 1 INTRODUCTION

As one of the most common malignancies, Hepatocellular carcinoma (HCC) has high incidence and mortality rates worldwide.^1^ To date, radical surgical resection is a well‐recognized curative therapy to treat HCC.^2^, ^3^ Although surgical resection could prolong the overall survival, the five‐year survival rate of HCC patients with radical resection is still unsatisfactory due to the high recurrence rate.^4^ Current conventional predictive factors, such as prothrombin induced by vitamin K absence‐II (PIVKA‐II) and serum alpha‐fetoprotein (AFP), are poor at predicting the prognosis of HCC.^5^, ^6^, ^7^ Therefore, it is urgently needed to identify new factors for predicting the recurrence of high‐risk patients, which would be valuable for stratifying the postoperative patients and enabling appropriate individualized therapeutic strategies.

Natural killer (NK) cells are important effector cells in the innate immune system, which are the first defense line of the body against the invasion of pathogens and tumor cells, and perform an important biological function in immune clearance and immune surveillance.^8^ NK cells are abundant in human liver, accounting for 30% ~ 50% of intrahepatic lymphocytes, which is 2–5 times higher than that in peripheral blood. The cytokine production and cytotoxicity of liver‐resident NK cells were higher than those of NK cells in peripheral blood, and some of them had unique immunophenotypes and functional characteristics.^9^ The C‐type lectin receptor NKG2D, the activating receptor of NK cells, can recognize stress‐induced ligands with different affinities, such as MHC class I chain‐related proteins and UL16‐binding proteins families (e.g., MICA, MICB, and ULBP1‐6).^10^ NKG2D ligands were frequently expressed in virus‐infected or stressed cells, and malignant cells, rather than normal cells.^11^ Thus, the interaction between the activating receptor and ligands of NKG2D would enhance the antitumor immune response of tumor‐infiltrating NK cells.

Compelling evidence has shown that NKG2D ligands were aberrantly expressed in HCC and associated with HCC prognosis.^12^, ^13^, ^14^ In 2012, Kamimura et al. identified that ULBP1 was mainly expressed in dysplastic nodule (DN), well and moderate‐differentiated HCC, rather than in poor‐differentiated HCC. Moreover, HCC patients with positive ULBP1 have longer recurrence‐free survival (RFS) compared with patients with negative ULBP1.^15^ However, Cadoux et al. indicated that ULBP1 was expressed highly in HCC, which was correlated with low levels of differentiation. Moreover, HCC patients with high ULBP1 were prone to early recurrence and had a poor prognosis.^16^ Thus, the relationship between NKG2D ligands expression and HCC recurrence remains controversial.

In this study, we determined all NKG2D ligands expression in HCC and evaluated their association with HCC prognosis or clinicopathological factors. In addition, the signature based on NKG2D ligands was constructed for predicting HCC recurrence.

## 2 MATERIALS AND METHODS

### 2.1 Data collection

TCGA database (TCGA‐LIHC) including transcriptome sequencing data (50 normal tissues and 374 tumor samples) and clinical information till February 21, 2021, were used as the training cohort (https://portal.gdc.cancer.gov/). Validation cohort containing 159 HCC patients with transcriptome sequencing and clinical data derived from the OEP000321 in NODE (https://www.biosino.org/node).^17^ A total of 231 paired HCC and adjacent tissues with corresponding clinical data were randomly collected between January 1, 2006, and December 31, 2016, from the Affiliated Hospital of Guilin Medical University (Guilin cohort). And the patients involved in this study underwent auxiliary examinations before surgery and postoperative pathologically examined confirmed as HCC. The main exclusion criteria were as follows: (a) history of other cancers, (b) death in 2 months after surgery, (c) incomplete follow‐up or clinical data. These tissue samples were subjected to immunohistochemistry, tissue microarray, and quantitative real‐time polymerase chain reaction (QRT‐PCR). While the written informed consent was signed by each participant.

### 2.2 Development and validation of a prognostic NKG2D ligands gene signature

Multivariate Cox analysis of RFS was used to determine NKG2D ligands gene with prognostic values in the training cohort. The prognostic NKG2D ligands gene was identified with the cutoff value of *p* < 0.05. To further evaluate the performance of NKG2D ligands gene signature, HCC patients in the two cohorts were classified into low‐risk and high‐risk groups based on the median expression of gene signature. RFS curves based on the Kaplan–Meier method were plotted via survminer package, and receiver operating characteristic curve (ROC) (1‐, and 3‐year RFS) was plotted by timeROC R package while Calibration curves were plotted by rms package.

### 2.3 Clinical correlation analysis and immune cell infiltration analysis

The correlation between NKG2D ligands gene signature and clinical‐pathological characteristics was analyzed and plotted by GraphPad Prism 8.0.2. Immune cell infiltration from transcriptome data was analyzed by the CIBERSORT tool (https://cibersortx.stanford.edu/).

### 2.4 Tissue microarray and immunohistochemistry

Tissue microarray including 137 paired HCC tissues and para cancer tissues was subjected to immunohistochemistry (IHC). Antibodies used in IHC were as follows: goat anti‐human ULBP‐2/5/6 (Invitrogen Cat# PA5‐47118, 1:500 dilution), rabbit anti‐human MICA/MICB (Abcam Cat# ab224702, 1:50 dilution), rabbit anti‐human ULBP1 (Abcam Cat# ab238331, 1:1000 dilution), rabbit anti‐human ULBP3 (Novus Cat# nbp2‐31,866, 1:1000 dilution), and mouse anti‐human ULBP4 (Santa Cruz Cat# sc‐390,784, 1:50 dilution). The details of the IHC assay were performed as previously described.^18^

The stained tissue microarray was scored by two pathologists on the following criteria: (I) the staining intensity: 0 (negative, no staining), 1 (weak, faint yellow staining), 2 (moderate, yellow staining), and 3 (strong, brown staining); (II) the percentage of positive cells: 0 (0%), 1 (1%–10%), 2 (11%–50%), 3 (51–80%), and 4 (80%–100%). The IHC score of NKG2D ligands expressed in HCC was calculated as follows: staining intensity score × the percentage score of positive cells, and graded as follows: (−), 0 points; (+), 1–3 points; (++), 4–6 points; and (+++), 7–12 points.

### 2.5 Quantitative real‐time polymerase chain reaction

Total RNA of paired fresh‐frozen HCC and adjacent normal liver tissue samples was extracted by the RNeasy Mini Kit (Qiagen), and the Agilent 2100 Bioanalyzer (Agilent Technologies) was used to detect the RNA quality of the samples. The amplified PCR fragments of MICA, ULBP3, and ULBP5 were analyzed with Sanger sequencing (Figure S1). The mRNA expression of MICA, ULBP3, and ULBP5 in the Guilin cohort was determined by QRT‐PCR, which was performed as previously described.^16^ The primer sequences for QRT‐PCR were in Table 1. The relative expression of MICA, ULBP3, and ULBP5 was calculated and normalized against the expression of GAPDH.

**TABLE 1:** The primer sequences used for QRT‐PCR

Gene	The primer sequence	The size
MICA	Forward: GAATCCGGCGTAGTCCTGAG	139 bp
	Reverse: CCTGACGCCAGGTCAGTATG	
ULBP3	Forward: TGGTCTCAATGAGAGACTGC	119 bp
	Reverse: TATGGCTTTGGGTTGAGCTAA	
ULBP5	Forward: CATGTGTCTCCTCATATGCTC	190 bp
	Reverse: TAAAGTCACGCGAGTCACG	
GAPDH	Forward: GTCTTCACCACCATGGAGAAG	225 bp
	Reverse: CATGAGTCCTTCCACGATACC	

### 2.6 Statistical analysis

Continuous variables following with normal distribution were shown as mean ± standard deviation (SD) and analyzed by Student's *t*‐test or Wilcoxon signed‐rank test. Categorical variables were analyzed via Chi‐square tests or Fisher exact test. Survival curves were plotted by Kaplan Meier methods and compared in the Log‐rank test. Multivariate analyses were determined by the Cox regression analysis. Statistical analysis was performed by SPSS18.0 (SPSS Inc.) and R (version 4.0.3 https://www.rproject.org/), and *p* < 0.05 was considered statistically significant.

The risk score was calculated by a formula below:

$$\begin{array}{c} \text{Risk score}={\text{coefficient1}}^{*}\;\text{gene1}+\ldots +{\text{coefficientN}}^{*}\;\text{geneN},\\ \text{in which the coefficient was determined}\;\mathrm{by}\;\text{predict}.\text{coxph}\\ \left(\text{type}={}^{'}{\text{risk}}^{'}\right)\;\text{function of survival package},\\ \text{while Hazard ration}=e^{\text{coefficient}}. \end{array}$$

## 3 RESULTS

### 3.1 The baseline characteristics of HCC cohorts

The datasets from TCGA‐LIHC, OEP000321, and Guilin cohort were involved in this study. And the clinical characteristics of HCC patients are shown in Table 2. As shown in the table, the majority of HCC patients were male and had no lymph node metastasis among the three cohorts. There were more G2 stage patients in TCGA‐LIHC cohort. In OEP000321 and Guilin cohort, most patients had liver cirrhosis. In this article, TCGA‐LIHC cohort was set as the training cohort, OEP000321 and Guilin cohorts were set as the validation cohort.

**TABLE 2:** The clinicopathological characteristics of HCC patients based on the TCGA‐LIHC, OEP000321 and Guilin cohort

Clinical characteristics	TCGA‐LIHC cohort (*N* = 374)	OEP000321 cohort (*N* = 159)	Guilin cohort (*N* = 137)
age (<55/≥55)	119/255	84/75	95/42
gender (male/female)	253/121	128/31	120/17
Liver cirrhosis (present/absent)	NA	112/47	116/21
Tumor number (single/multiple)	NA	117/42	80/57
Tumor (T1/T2/T3/T4/TX/NA)	184/93/81/13/1/2	NA	NA
Tumor size (<5 cm/≥5 cm)	NA	63/96	19/118
Node (N0/N1/NX/NA)	256/4/113/1	157/2/0/0	125/10/2/0
Metastasis (M0/M1/MX)	272/3/99	NA	126/11/0
Tumor thrombus (present/absent)	NA	37/122	32/105
TNM (I/II/III/IV)	NA	91/14/52/2	NA
BCLC stage (A/B/C)	NA	68/52/39	NA
grade (G1/G2/G3/G4/NA)	55/180/121/13/5	NA	NA
stage (I/II/III/IV/NA)	174/86/86/4/24	NA	NA
Recurrence (present/absent/NA)	138/168/68	103/56/0	47/90/0

Abbreviation: NA, not applicable.

### 3.2 NKG2D ligands were differentially expressed between the adjacent non‐tumor and tumor tissues of HCC patients

**FIGURE 1:**
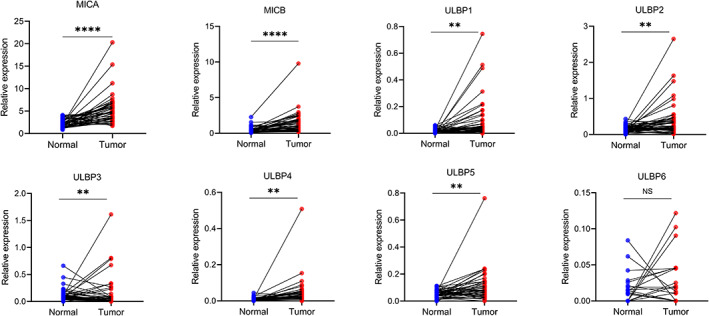
The mRNA expression of NKG2D ligands in paired HCC tumor and adjacent normal tissues from TCGA‐LIHC dataset. **p < 0.01, ****p < 0.0001.

In TCGA‐LIHC dataset, 50 paired tumors and adjacent normal tissues were selected to detect the NKG2D ligand expression. We found the mRNA expression level of MICA, MICB, ULBP1, ULBP2, ULBP4, and ULBP5 was significantly higher in tumor tissues than that in adjacent normal tissues, whereas ULBP3 expression was lower in tumor tissues compared to adjacent normal tissues. There was no significant difference in ULBP6 expression between normal and tumor tissues (Figure 1A).

**FIGURE 2:**
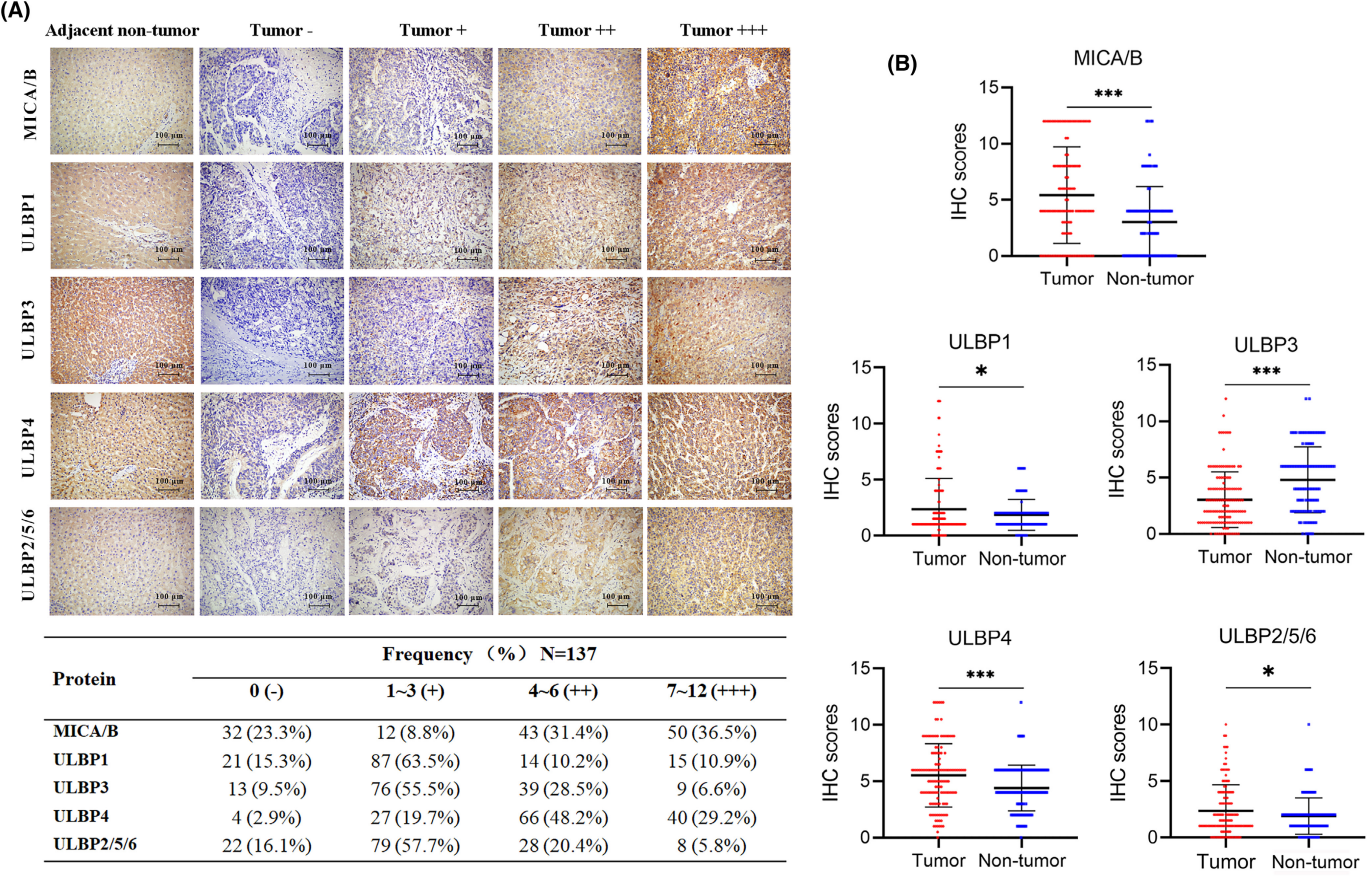
The protein expression of NKG2D ligands. (A) the distribution and frequency of NKG2D ligands expression in Guilin cohort via Immunohistochemistry (IHC) staining (−, negative expression; +, weak expression; ++, moderate expression; and +++, high expression). (B) NKG2D ligands expression was compared in tumor and adjacent normal tissue in the Guilin cohort via IHC analysis. *p < 0.05, ***p < 0.001.

In addition, we performed immunohistochemistry to determine the protein levels of NKG2D ligands in the tissue microarray from the Guilin cohort. We found that NKG2D ligands were heterogeneously expressed in the cytoplasm of normal and tumor cells. The protein expression pattern of MICA/B, ULBP1, ULBP3, and ULBP4 between paired tumor and adjacent tissues was consistent with that in mRNA levels of the TCGA‐LIHC dataset (Figure 2B). Importantly, the protein expression of ULBP2/5/6 was significantly higher in tumor tissues than that in adjacent normal tissues (Figure 2B). MICA/B and ULBP4 were moderately (IHC score: 4–6) or highly (IHC score: 7–12) expressed in more than 2/3 HCC tumor samples, whereas ULBP1, ULBP3, and UBP2/5/6 were not (IHC score: 0) or lowly (IHC score: 1–3) expressed in more than 2/3 HCC tumor samples (Figure 2A).

To estimate the significance of NKG2D ligands expression in HCC, the relationship was analyzed between NKG2D ligands expression level and clinicopathological variables of patients in the Guilin cohort (Table S1). Low expression of MICA/B was associated with large tumor size (*p* = 0.029) and the risk of portal vein tumor thrombus (PVTT) (*p* = 0.025). High expression of ULBP1 was associated with cirrhosis (*p* = 0.036). Low expression of ULBP3 was associated with high serum HBsAg level (*p* = 0.022) and tumor size (*p* < 0.001). Low ULBP4 expression was associated with tumor invasion and metastasis (*p* = 0.011). Low ULBP2/5/6 expression was associated with the advanced TNM stage (*p* = 0.031). There were no significant associations between NKG2D ligand expression and gender, age, family history, drinking, AFP, tumor number, or lymph node invasion.

### 3.3 The association between NKG2D ligands and prognosis

The prognostic significance of NKG2D ligands in HCC was confirmed in the TCGA‐LIHC dataset. We found that highly expressed MICA, MICB, ULBP2, ULBP4, and ULBP5 were all related to poor RFS, while the low expression of ULBP1, ULBP3, and ULBP6 was all associated with poor RFS (Figure S2).

### 3.4 The development and validation of a three‐NKG2D ligands signature to predict recurrence‐free survival

**FIGURE 3:**
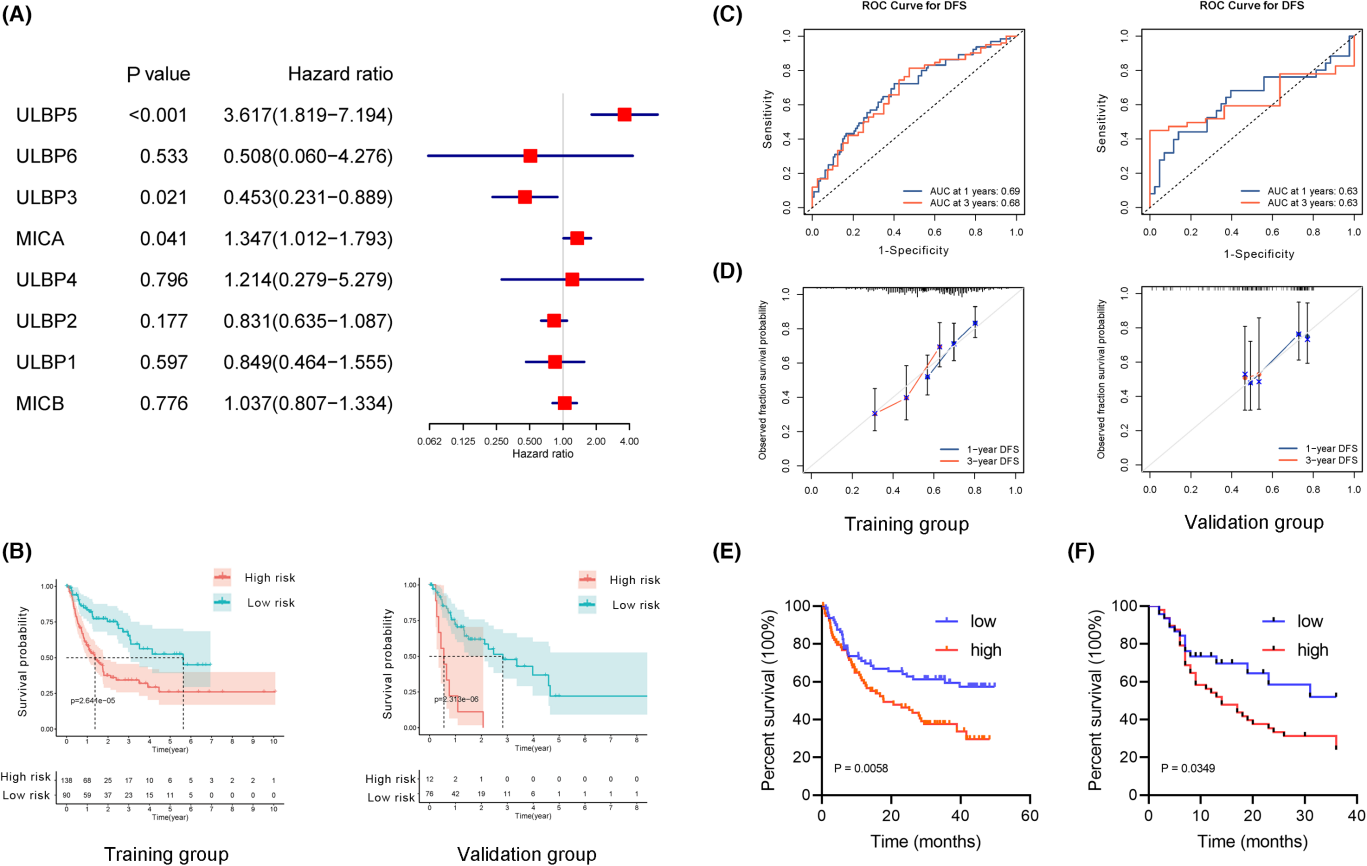
The predictive value of the three‐NKG2D ligands signature in training and validation cohorts. (A) Multivariate Cox regression analysis for HCC recurrence in TCGA‐LIHC training cohort. (B) Survival curves for RFS in training and validation group from TCGA‐LIHC dataset. (C) ROC curves to predict 1‐ and 3‐year RFS in training and validation group from TCGA‐LIHC dataset. (D) Calibration curves in training and validation group from TCGA‐LIHC dataset. (E) RFS curve of the three‐NKG2D ligands signature in OEP000321 dataset. (F) RFS curve of the three‐NKG2D ligands signature in Guilin cohort.

First of all, the TCGA‐LIHC cohort were divided into training and internal validation groups at the ratio of 7:3. Multivariate analysis by Cox regression revealed the expression levels of MICA (HR = 1.347; 95% CI = 1.012–1.793; *p* = 0.041), ULBP3 (HR = 0.453; 95% CI = 0.231–0.889; *p* = 0.021) and ULBP5 (HR = 3.617; 95% CI = 1.819–7.194; *p* < 0.001) were associated with RFS (Figure 3A). Then, the correlation coefficients of the above three NKG2D ligands were determined and the risk score of each HCC patient was calculated according to the following formula: Risk score = 0.29795 × (MICA) ‐ 0.79165 × (ULBP3) + 1.28571 × (ULBP5). HCC patients were divided into low‐ and high‐risk groups according to the median risk score. In the TCGA dataset as the training cohort, HCC patients in the high‐risk group had shorter RFS than those in the low‐risk group (Figure 3B). In addition, ROC curves indicated area under the curves (AUC) for 1‐ and 3‐year RFS were 0.69 and 0.68 in the training group while 0.63 and 0.63 in the validation group, respectively (Figure 3C). The calibration curves showed good consistency between predicted and observed survival probabilities (Figure 3D). Overall, these results revealed that the three‐NKG2D ligands signature had a good performance in predicting RFS.

Furthermore, the OEP000321 dataset and Guilin cohort as validation cohorts were subjected to verify the effectiveness of this signature. First, we observed that MICA and ULBP5 were significantly higher in HCC tumor tissues than those in paired normal tissues, while ULBP3 was lower in tumor tissues compared to normal tissues in the OEP000321 dataset (Figure S3) and Guilin cohort (Figure S4). We also observed that patients with high MICA and ULBP5 expression were both correlated with poor RFS, while patients with low ULBP3 expression were correlated with poor RFS (Figures S3 and S4). The results were consistent with those of TCGA‐LIHC cohort (Figure 1, Figure S2). Next, the survival curves showed poor RFS in the high‐risk group in OEP000321 dataset (Figure 3E) and Guilin cohort (Figure 3F), in accord with the result of the training cohort. These results suggested the good predictive value of the three‐NKG2D ligands signature for RFS.

### 3.5 The associations between the three‐NKG2D ligands signature and clinicopathological characteristics

**FIGURE 4:**
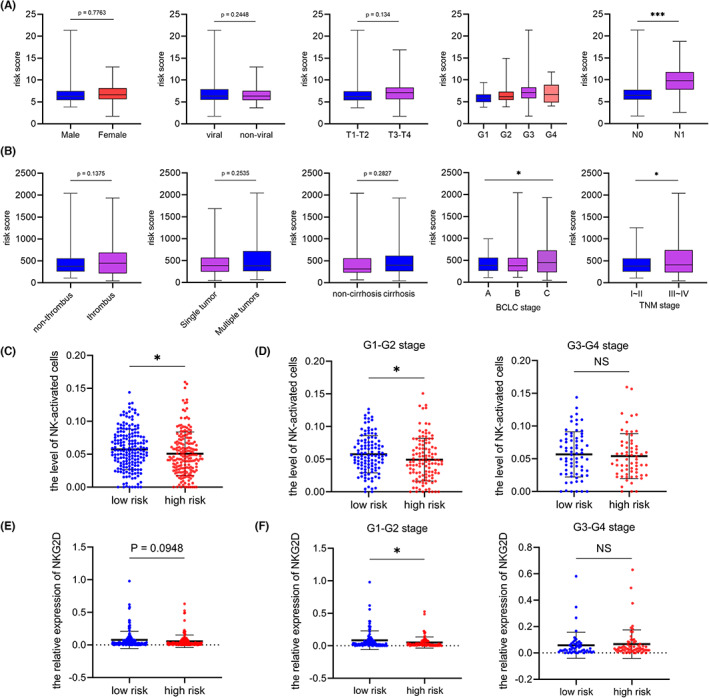
Clinical correlation analysis of the three‐NKG2D ligands signature. (A) Correlations between the three‐NKG2D ligands signature and clinical characteristics in TCGA‐LIHC dataset. (B) Correlations between the three‐NKG2D ligands signature and clinical characteristics in the OEP000321 dataset. (C) NK‐activated cell infiltration in low‐risk and high‐risk subgroups via CIBERSORT in TCGA‐LIHC dataset. (D) NK‐activated cell infiltration in low‐risk and high‐risk subgroups via CIBERSORT in TCGA‐LIHC G1‐G2 and G3‐G4 stages. (E) The expression of NKG2D in low‐risk and high‐risk groups in TCGA‐LIHC dataset. (F) The NKG2D expression in low‐risk and high‐risk subgroups compared in TCGA‐LIHC G1‐G2 and G3‐G4 stages. *p < 0.05, ****p < 0.0001.

In order to explore the clinical significance of the three‐NKG2D ligands signature in HCC, we analyzed the correlations between the signature and clinicopathological features. In the TCGA‐LIHC dataset, the high‐risk patients were prone to lymph node metastasis compared to low‐risk patients; However, there were no correlations between the signature and gender, viral infection, or T stage (Figure 4A). In the OEP000321 dataset, patients with high TNM and BCLC stages showed high‐risk values. However, no significant correlations were found between the signature and PVTT, tumor number, or cirrhosis (Figure 4B).

**FIGURE 5:**
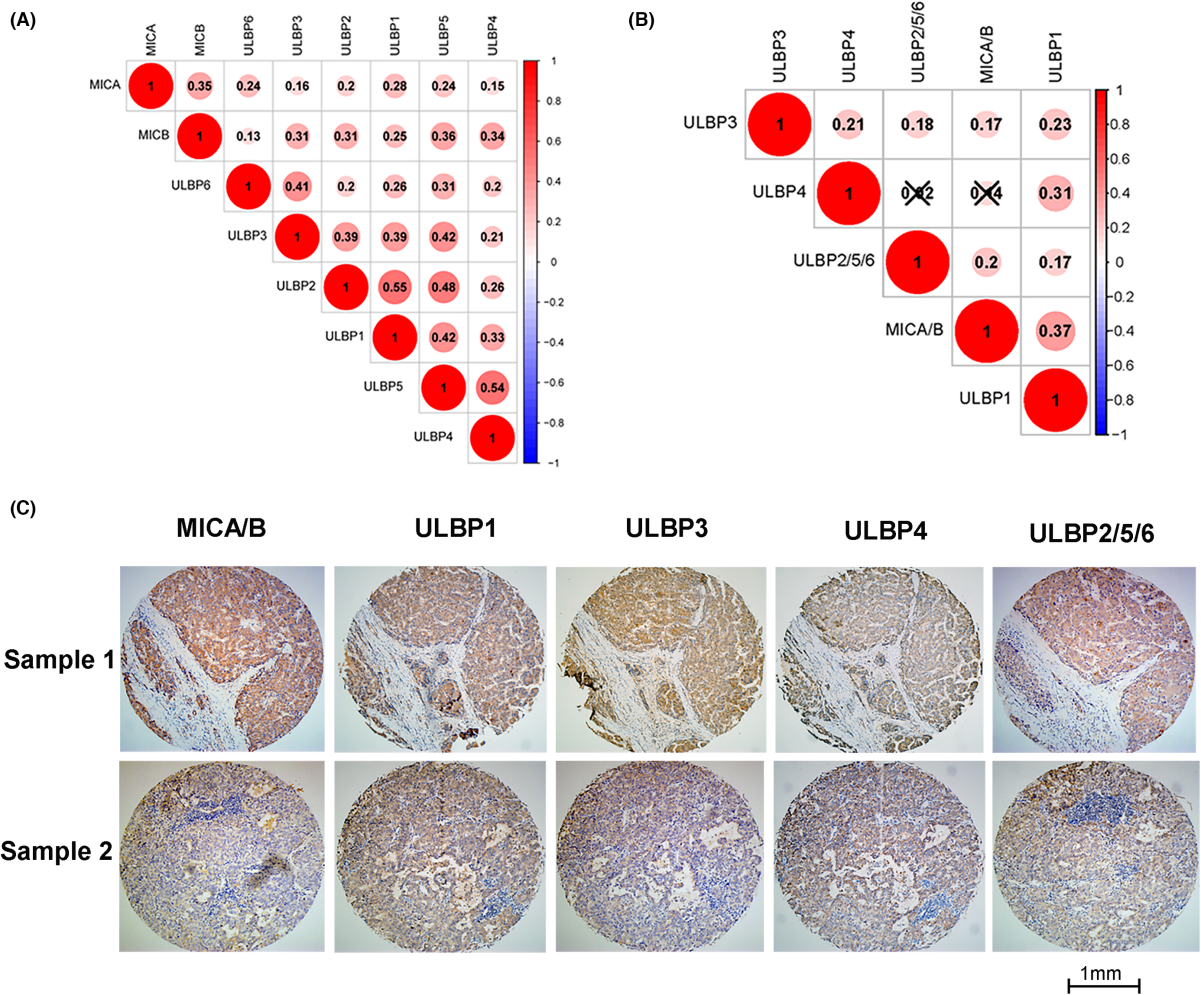
Correlation analysis among NKG2D ligands. (A) Correlations among NKG2D ligands in TCGA‐LIHC dataset. (B) Correlation among NKG2D ligands in the OEP000321 dataset. (C) Immunohistochemistry of the NKG2D ligands in two typical samples.

Due to the vital function of NKG2D ligands in tumor immunity, we speculated the three‐NKG2D ligands signature might play a critical role in HCC immune microenvironment. Therefore, we explored the immune cell subtypes in HCC samples via CIBERSORT in the TCGA‐LIHC dataset and found the signature was associated with the population of NK‐activated cells (Figure 4C). Interestingly, the infiltration level of NK‐activated cells in high‐risk patients was lower than that in low‐risk patients with G1‐G2 stage (Figure 4D), while there was no significance in G3‐G4 stage patients (Figure 5E). Further, we also examined the correlations between the signature and NKG2D (Figure 4E) and observed that compared to the low‐risk patients with G1‐G2 stage, the level of NKG2D expression was lower in the high‐risk patients, but there was no difference in patients with G3‐G4 stage (Figure 4F).

In order to explore the possible mechanisms of HCC recurrence, the enrichment analysis was performed between high‐risk and low‐risk patients in the TCGA‐LIHC dataset. GO analysis suggested that cadherin binding in molecular function was enriched (Figure S5). The loss of function of cadherin is thought to contribute to cancer progression by increasing proliferation, invasion, or metastasis,^5^, ^19^ which might be involved in HCC recurrence between the two groups. KEGG analysis showed that viral carcinogenesis played an important role between the two groups (Figure S6),^11^ which might affect the expression and function of NKG2D ligands in HCC.

### 3.6 The associations among NKG2D ligands in HCC

Firstly, the correlations among the NKG2D ligands were analyzed in HCC cohorts. In the TCGA‐LIHC dataset, the eight NKG2D ligands were highly correlated with each other (Figure 5A). In the OEP000321 dataset, there were positive correlations between MICA and MICB, ULBP1 and ULBP2, ULBP1 and ULBP3, ULBP2 and ULBP3, ULBP2 and ULBP5, or ULBP4 and ULBP5 (Figure S7). In the Guilin cohort, except for ULBP4 and MICA/B, ULBP4, and ULBP2/5/6, an obvious correlation was observed among the NKG2D ligands (Figure 5B,C). In the three cohorts, ULBP3 was significantly correlated with other NKG2D ligands.

## 4 DISCUSSION

Various risk factors lead to the occurrence of HCC, such as hepatitis B virus (HBV) infection, hepatitis C virus (HCV) infection, alcohol (ethanol), non‐alcoholic fatty liver disease (NAFLD), carcinogen/toxin exposure, genetic factors, etc.^19^ The expression of the NKG2D ligands might serve as indicators of cellular stress, which could be induced by the viral infection or malignant transformation.^20^ Accumulating evidence demonstrates that the NKG2D ligands are expressed in multiple malignant tumors, especially in HCC.^11^ Currently, NKG2D ligands have become a crucial target for cancer management. Thus, it is important to confirm the expression and distribution of all NKG2D ligands in HCC. In this paper, a comprehensive analysis of the eight NKG2D ligands expression was conducted in HCC tissues. Interestingly, in the TCGA‐LIHC and Guilin cohort, there was a significant difference in the expression of MICA, MICB, and ULBP1‐5 between HCC tumor and adjacent non‐tumor tissues. Moreover, compared with other ligands, MICA/B and ULBP4 are moderately or highly expressed in more than 2/3 of HCC patients, which supported that the NKG2D ligands might serve as therapeutic targets.

Liver resection remains the most efficient treatment for HCC patients, but the postoperative recurrence rate is high which would eventually lead to a poor prognosis.^4^, ^21^ AFP is related to the HCC occurrence and progression and widely used as an indicator of HCC diagnosis and prognosis. However, for nearly 30% of HCC patients with negative AFP, there is a lacking of effective biomarkers.^22^ Hence, it is urgently needed to find novel biomarkers to predict the recurrence of HCC patients after surgery. Previous studies have revealed the relationship between the NKG2D ligand and prognosis, but the prognostic values were still controversial in different cohorts.^15^, ^16^ We confirmed in this study multiple NKG2D ligands were differently expressed in HCC, therefore, there might be a higher predictive value of the signature based on the multiple NKG2D ligands. Intriguingly, MICA, ULBP3, and ULBP5 were shown closely related to the recurrence of HCC. A signature was developed based on the expression of the three NKG2D ligands above in TCGA‐LIHC dataset. We have also validated the prediction signature in OEP000321 and Guilin datasets. We further analyzed the correlation between signature and clinical characteristics and found that the risk score of the signature was not significantly different between virus‐infected and non‐virus‐infected HCC patients (Figure 4A), which suggested that the prediction signature was not affected by the factor of virus infection, and may better reflect the signature's generalization capacity. However, we found that the high‐risk score based on the signature is significantly correlated to lymph node metastasis, BCLC stage, and TNM stage, which suggested that HCC patients with high risk indicated a more aggressive phenotype.

Since NK cells expressing NKG2D could trigger anti‐tumor immunity by recognizing NKG2D ligands,^23^ we evaluated the immune infiltration of each patient in the TCGA‐LIHC dataset by the CIBERSORT tool and identified there was a significant difference in the level of NK cells infiltration between low and high subgroups. Importantly, the levels of infiltrated NK‐activated cells and NKG2D expression in HCC patients were both lower in the high‐risk group compared to the low‐risk group with the G1‐G2 stage. Several studies have proved that tumor‐derived NKG2D ligands could obstruct the NKG2D pathway to restrain NK cytotoxicity.^11^, ^14^ These results suggest that NKG2D ligands in high‐risk patients with poorly differentiated HCC tumors might decrease the NKG2D expression in impaired NK cells, which need further experimental verification.

Most previous studies have mainly focused on the mechanistic roles of MICA, MICB, ULBP1, and ULBP2 in HCC,^13^, ^14^, ^16^ but few studies focused on other NKG2D ligands. We found ULBP3 expression was significantly correlated with other NKG2D ligands in the three datasets, which suggested that ULBP3 might play a crucial role in the HCC progression by regulating other NKG2D ligands. Moreover, the expression of ULBP3 in HCC tumor tissues was much lower than that in adjacent tissues, and patients with low ULBP3 had a high level of serum HBsAg (Table S1) and poor RFS. Notably, ULBP3 expression was positively correlated with the number of NK cells (Figure S8). It has been confirmed that HBsAg inhibited the expression of MICA and MICB by inducing cellular miRNAs in HCC.^24^, ^25^ These results suggested HBsAg might also inhibit the expression of ULBP3 in HCC cells, thus providing an opportunity for tumor immune escape. However, we need further studies to identify this perspective.

Compelling evidence has demonstrated the key role of the NKG2D/NKG2D‐ligand pathway in NK‐mediated cytolysis of HCC cells.^26^, ^27^ However, with the HCC progression, tumor cells could down‐regulate the NKG2D receptor expression in NK cells through multiple pathways.^14^, ^28^ Thus, the NKG2D impairment in HCC patients may be one of the important mechanisms of NK‐cell dysfunction. Therefore, maximizing the activation of NKG2D expression in NK cells and fully inducing NKG2D ligands expression in tumor cells has become a popular concept of cancer immunotherapy.^11^, ^26^ At present, researchers have constructed NKG2D‐CAR‐NK cells based on chimeric antigen receptor (CAR) technology using NKG2D as a signaling molecule, which could transmit signals to intracellular mediating cytotoxicity via CD3ζ and DAP10.^29^, ^30^ Compared with endogenous NK cells, NKG2D‐CAR‐NK cells can significantly enhance the activity of NKG2D and effectively kill various cancer cells expressing NKG2D ligands.^31^, ^32^, ^33^ In this study, MICA in HCC was highly expressed in the three datasets. These results suggest that the construction of NKG2D‐CAR‐NK cells targeting MICA with high affinity is expected to be a useful weapon for anti‐HCC therapy.

There are a few limitations in this study. Unlike the TCGA‐LIHC cohort as training cohort, the validation cohorts (OEP000321 dataset and Guilin cohort) in this study were both from China and most HCC patients were with HBV‐positive background. Though the predictive signature was not affected by the factor of virus infection, further study is needed to explore the mechanism of NKG2D ligands expression in HCC induced by virus, especially HBV.

In conclusion, we comprehensively studied the expression and distribution of the eight NKG2D ligands in HCC. MICA, MICB, ULBP1, ULBP4, and ULBP5 are highly expressed in HCC tissues and ULBP3 may perform an important function in the HCC progression. Importantly, the RFS prediction signature constructed on MICA, ULBP3, and ULBP5 could predict HCC recurrence in patients after hepatectomy and might provide a benefit in the management of postoperative patients as a valuable prediction tool.

## AUTHOR CONTRIBUTIONS

**Dongbo Chen:** Funding acquisition (equal); methodology (equal); writing – original draft (equal). **Jie Gao:** Formal analysis (equal); validation (equal). **Liying Ren:** Software (equal); visualization (equal). **Pu Chen:** Investigation (equal). **Yao Yang:** Validation (equal). **Shaoping She:** Validation (equal). **Yong Xie:** Conceptualization (equal). **Weijia Liao:** Data curation (equal); resources (equal); supervision (equal). **Hongsong Chen:** Conceptualization (equal); funding acquisition (equal); project administration (equal); writing – review and editing (equal).

## FUNDING INFORMATION

This research was funded in part by the National Key Sci‐Tech Special Project of China (grant number 2018ZX10302207), the Peking University Medicine Seed Fund for Interdisciplinary Research supported by “the Fundamental Research Funds for the Central Universities” (grant number BMU2021MX007 and BMU2022MX001), and the Peking University People's Hospital Scientific Research Development Funds (grant number RDX2020‐06), and Qi‐Min Project.

## CONFLICT OF INTEREST

The authors have no conflict of interest.

## ETHICS STATEMENT

The study was approved by the Ethics Committee of the Affiliated Hospital of Guilin Medical University and Peking University People's Hospital.

## Supporting information


Figure S1
Figure S2
Figure S3
Figure S4
Figure S5
Figure S6
Figure S7
Figure S8
Table S1

## Data Availability

I confirm that my article contains a Data Availability Statement even if no data is available (list of sample statements) unless my article type does not require one. I confirm that I have included a citation for available data in my references section, unless my article type is exempt.
