# Supplementary material for: A signature based on NKG2D ligands to predict the recurrence of hepatocellular carcinoma after radical resection
Source: Cancer Med. 2022 Oct 9;12(5):6337–47. doi: 10.1002/cam4.5318 (PMC10028019; doi:10.1002/cam4.5318)
Supplement: Supplementary file 1 — Figure S1 Figure S2 Figure S3 Figure S4 Figure S5 Figure S6 Figure S7 Figure S8 Table S1 [file CAM4-12-6337-s001.docx]

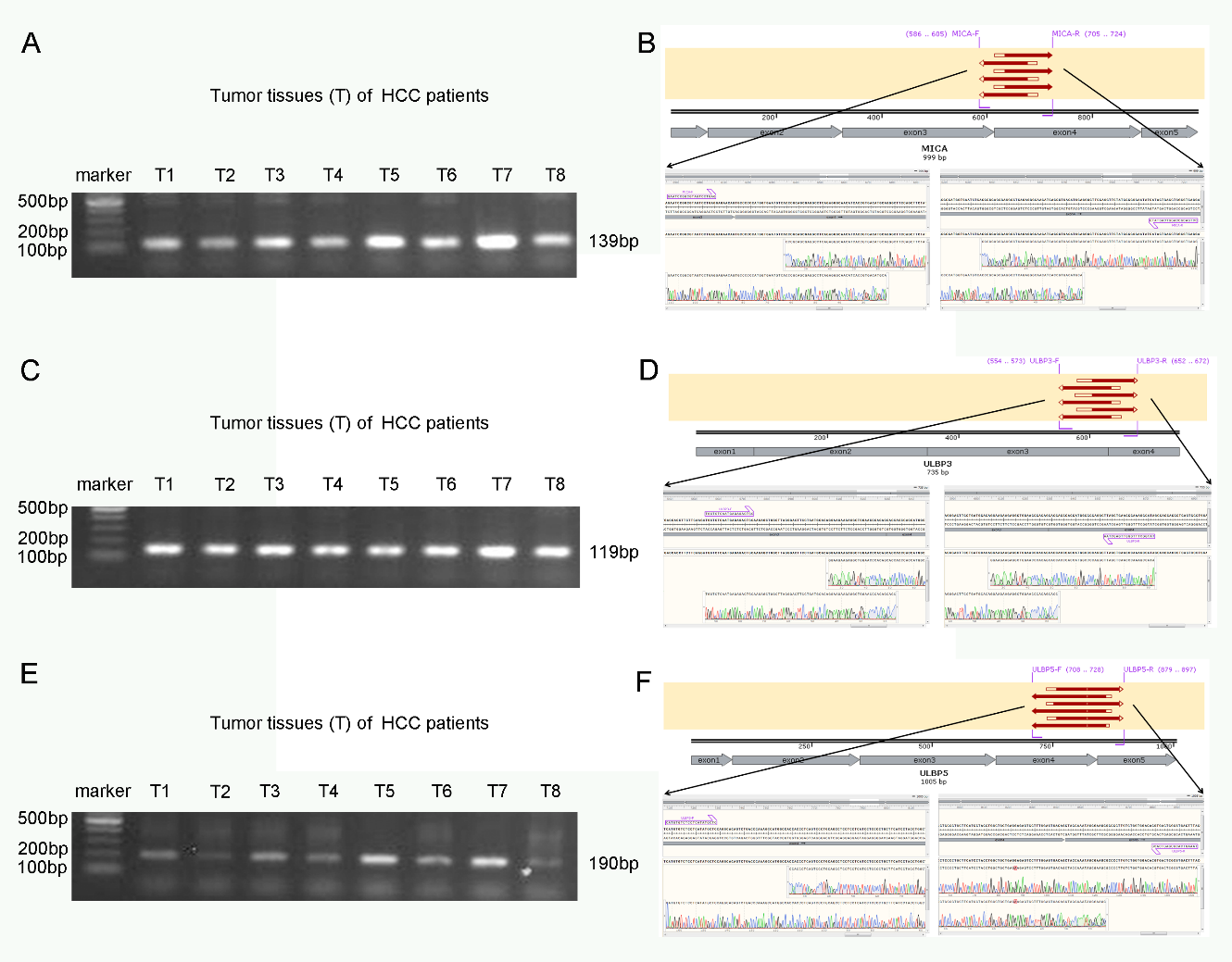


Figure S1. The mRNA expression of MICA, ULBP3, and ULBP5 determined by PCR and Sanger sequencing. (A) The mRNA expression of MICA in 8 HCC tumor tissues measured by PCR. (B) The amplified PCR fragment of MICA was analyzed with Sanger sequencing. (C) The mRNA expression of ULBP3 in 8 HCC tumor tissues measured by PCR. (D) The amplified PCR fragment of ULBP3 was analyzed with Sanger sequencing. (E) The mRNA expression of ULBP5 in 8 HCC tumor tissues measured by PCR. (F) The amplified PCR fragment of ULBP5 was analyzed with Sanger sequencing.


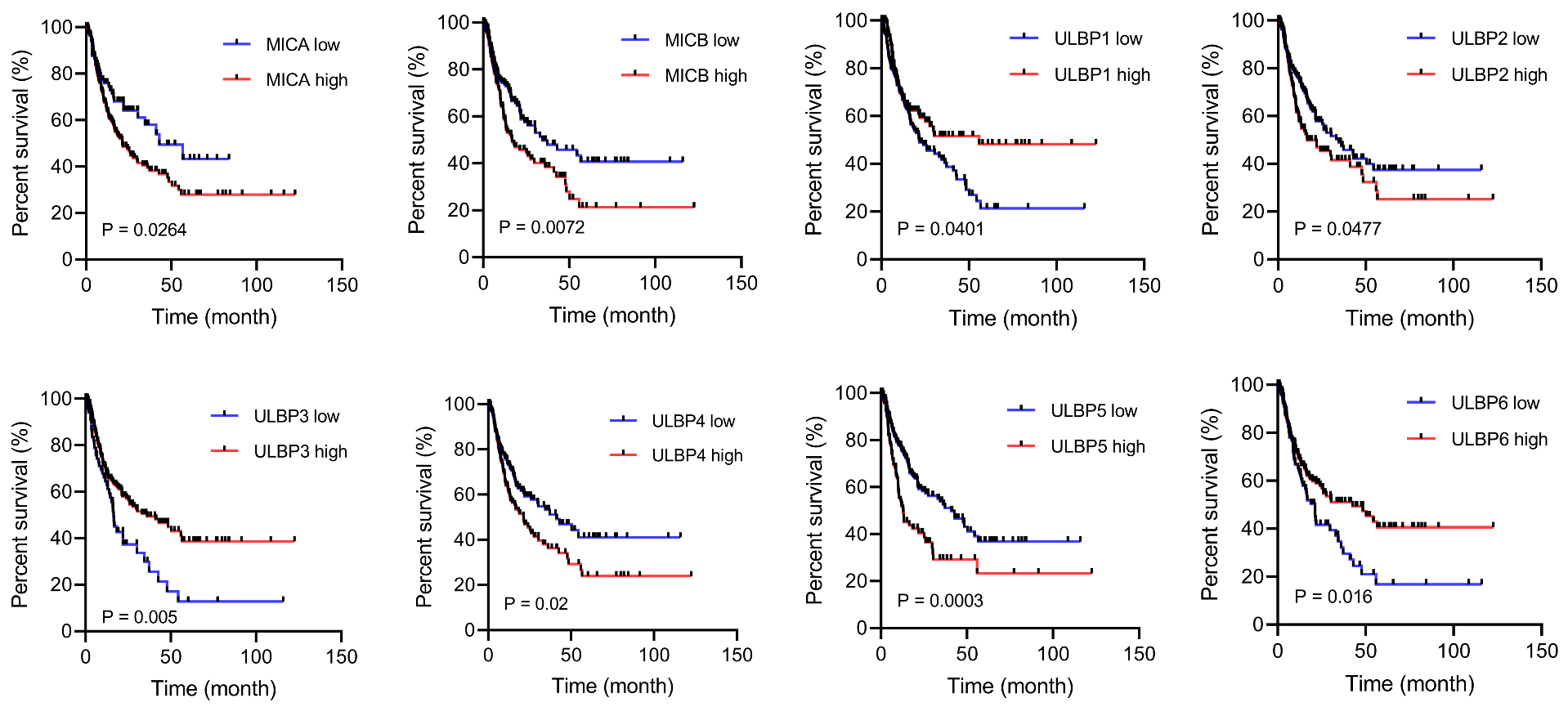


Figure S2. Recurrence-free survival (RFS) curves of the NKG2D ligands in TCGA-LIHC cohort.


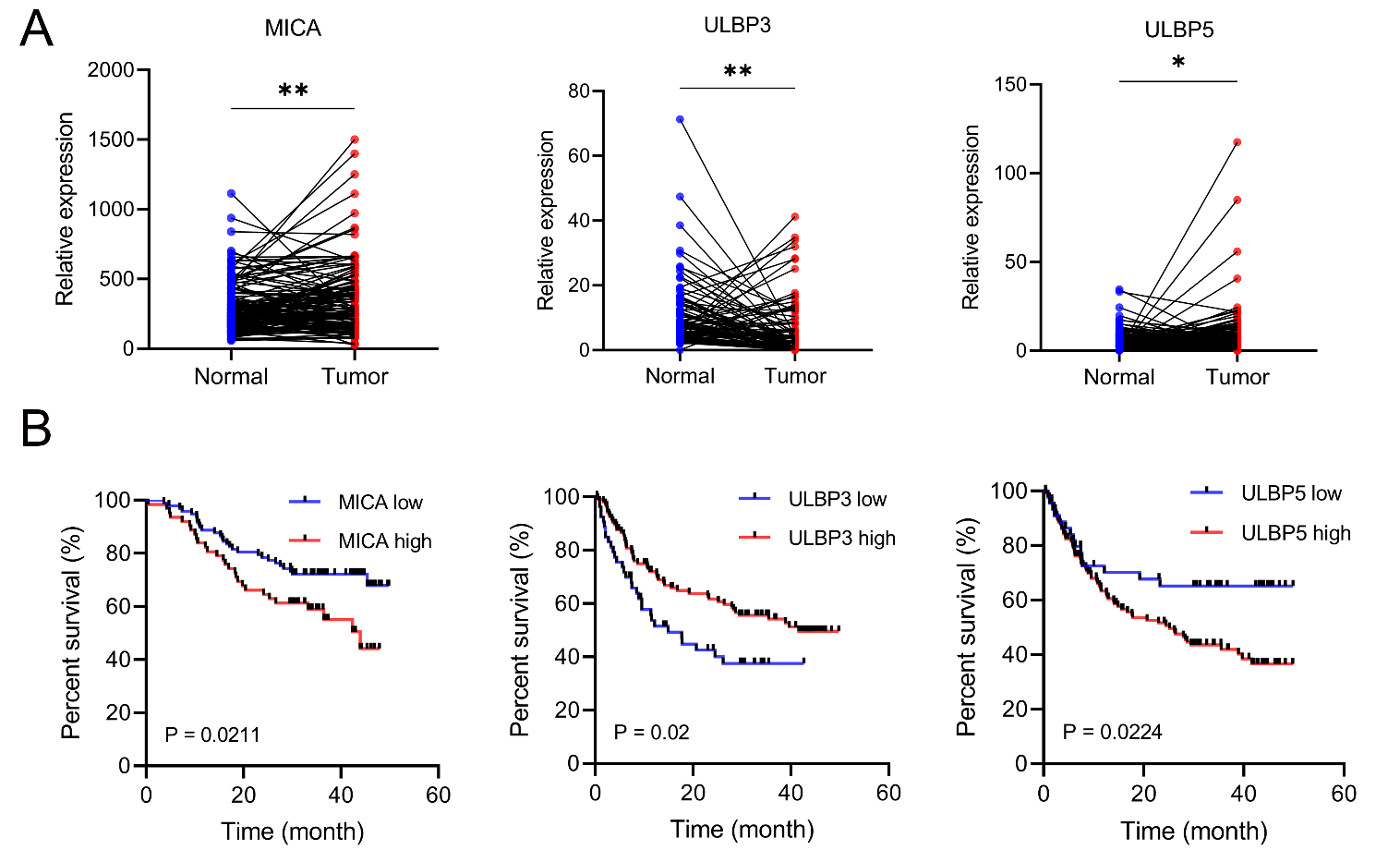


Figure S3. The expression and prognosis of NKG2D ligands in OEP000321 dataset. (**A**) The mRNA expression of NKG2D ligands in paired HCC tumor and adjacent normal tissues from OEP000321 dataset. (**B**) The recurrence-free survival (RFS) curves of the NKG2D ligands in OEP000321 dataset. *P < 0.05, **P < 0.01.


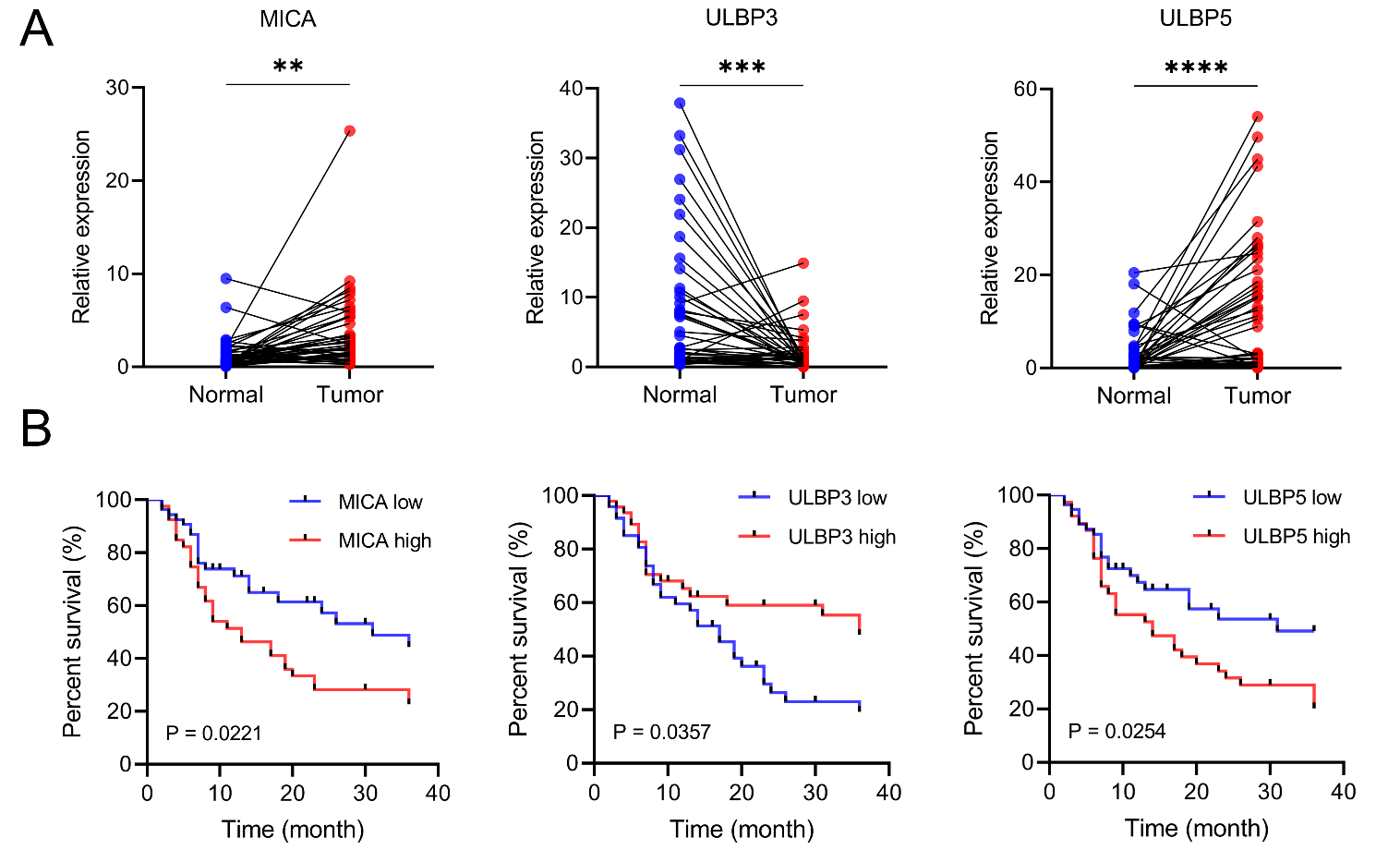


Figure S4. The expression and prognosis of NKG2D ligands in Guilin cohort. (**A**) The mRNA expression of NKG2D ligands in paired HCC tumor and adjacent normal tissues from Guilin cohort. (**B**) The recurrence-free survival (RFS) curves of the NKG2D ligands in Guilin cohort. **P < 0.01, ***P < 0.001, ****P < 0.0001.


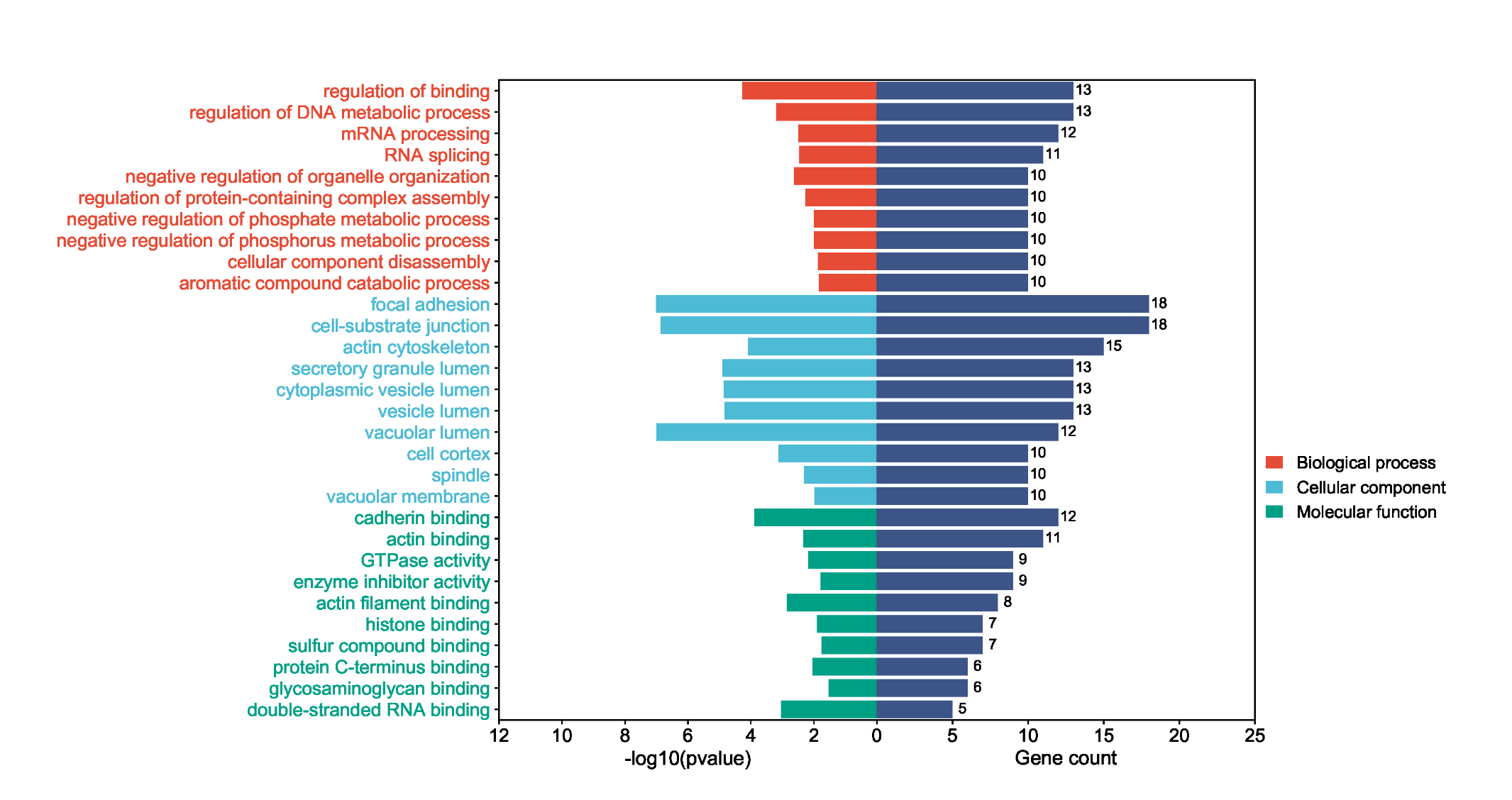


**Figure S5:** GO analysis performed between low- and high-risk patients in the TCGA-LIHC dataset.


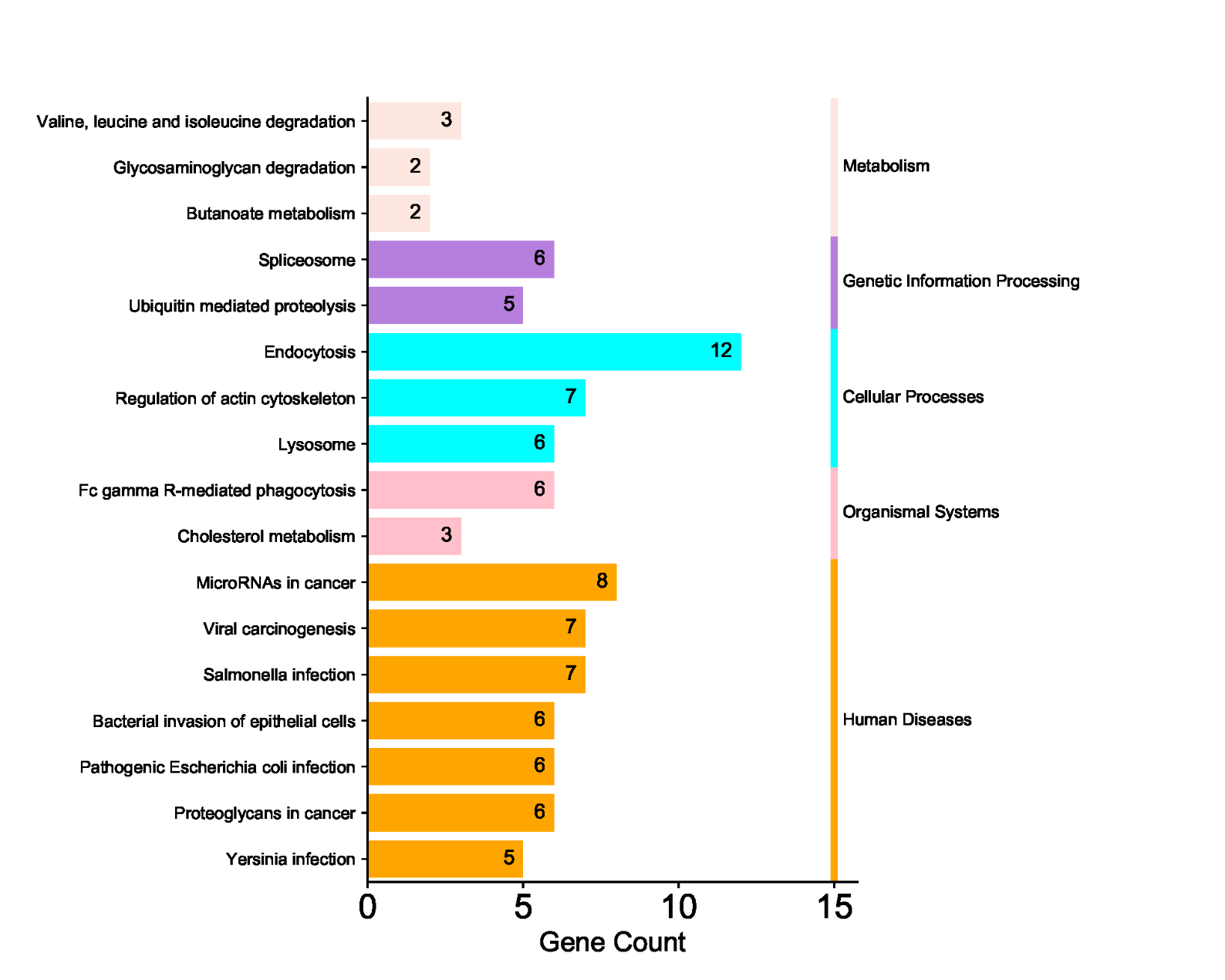


**Figure S6:** KEGG analysis performed between low- and high-risk patients in the TCGA-LIHC dataset.


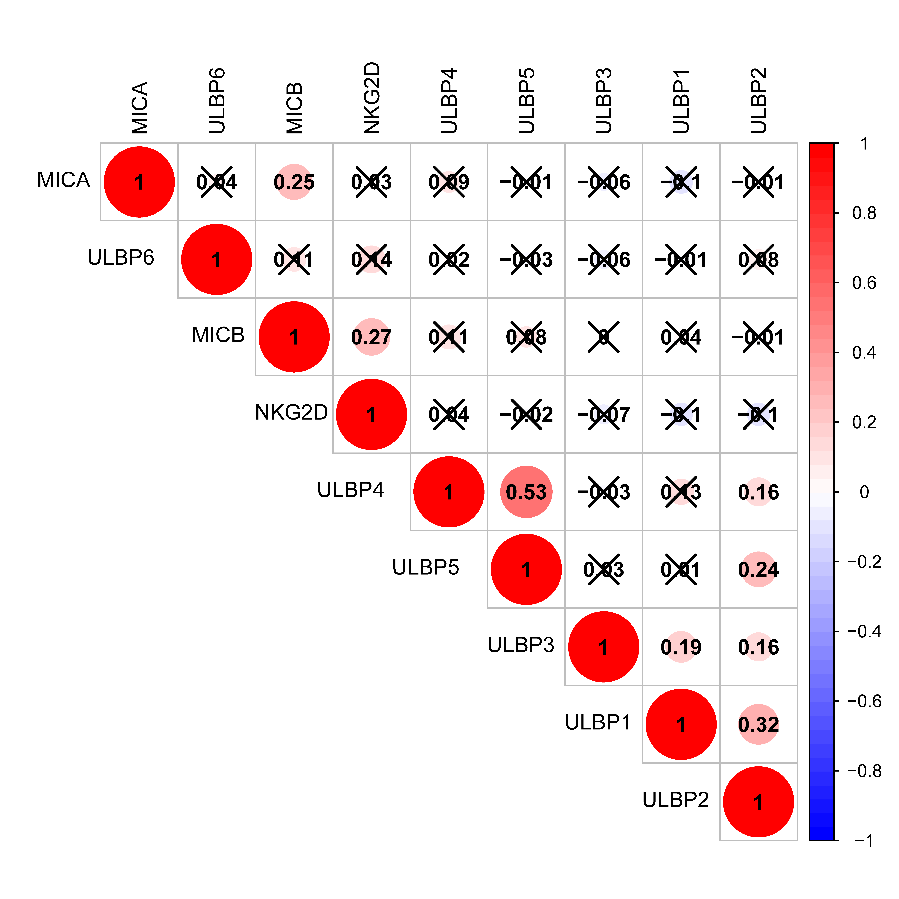


Figure S7. Correlation of NKG2D and its ligands in the OEP000321 dataset


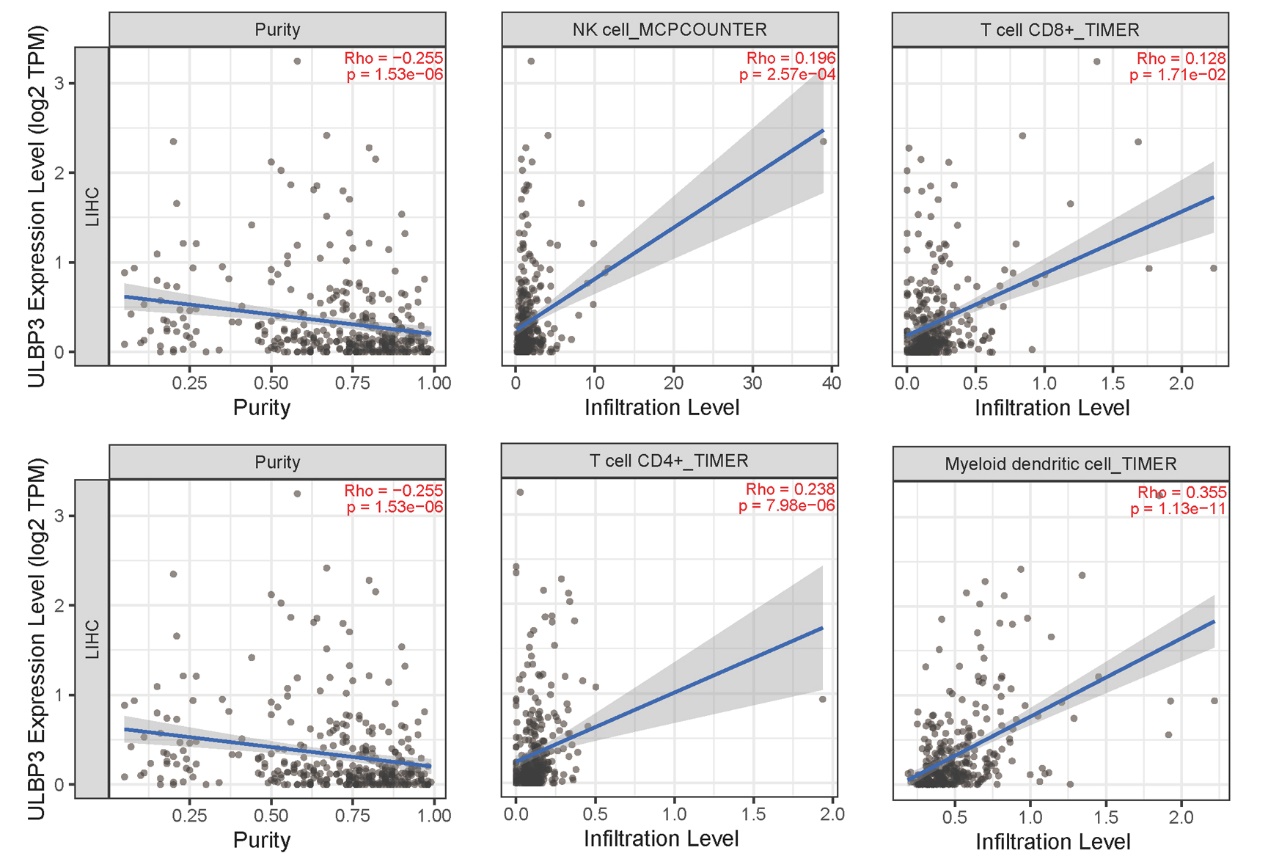


Figure S8. ULBP3 expression was positively correlated with the infiltration levels of NK cells, CD8^+^ T cells, CD4^+^ T cells and DC cells in the TCGA-LIHC dataset determined by the TIMER2 tool.

**Table S1**

**Table S1.** NKG2D ligands expression level and clinicopathological variables of patients in the Guilin cohort.

|  | **MICA/B expression** | | | **ULBP1 expression** | | | **ULBP3 expression** | | | **ULBP4 expression** | | | **ULBP2/5/6 expression** | | |
| --- | --- | --- | --- | --- | --- | --- | --- | --- | --- | --- | --- | --- | --- | --- | --- |
| **Parameter** | high | low | *P*-value | high | low | *P*-value | high | low | *P*-value | high | low | *P*-value | high | low | *P*-value |
|  | (n = 62) | (n = 75) |  | (n = 21) | (n = 116) |  | (n = 32) | (n = 105) |  | (n = 86) | (n = 51) |  | (n = 23) | (n = 114) |  |
| **Gender**  Female  Male | 9  53 | 8  67 | 0.496 | 3  18 | 14  102 | 0.777 | 1  31 | 16  89 | 0.121 | 10  76 | 7  44 | 0.719 | 4  19 | 13  101 | 0.487 |
| **Age**  <55  ≥55 | 43  19 | 52  23 | 0.998 | 16  5 | 79  37 | 0.460 | 26  6 | 69  36 | 0.095 | 61  25 | 34  17 | 0.601 | 16  7 | 79  35 | 0.980 |
| **Family history**  No  Yes | 51  11 | 57  18 | 0.372 | 17  4 | 91  25 | 0.796 | 22  10 | 86  19 | 0.111 | 67  19 | 41  10 | 0.731 | 16  7 | 92  22 | 0.233 |
| **Drinking**  No  Yes | 25  37 | 32  43 | 0.782 | 9  12 | 48  68 | 0.899 | 13  19 | 44  61 | 0.898 | 33  53 | 24  27 | 0.319 | 11  12 | 46  68 | 0.507 |
| **Cirrhosis**  No  Yes | 12  50 | 9  66 | 0.234 | 0  21 | 21  97 | **0.036*** | 6  26 | 16  89 | 0.636 | 15  71 | 7  44 | 0.567 | 2  21 | 19  95 | 0.333 |
| **HBsAg**  negative  positive | 16  46 | 14  61 | 0.315 | 2  19 | 24  92 | 0.365 | 12  20 | 19  86 | **0.022*** | 22  64 | 8  43 | 0.176 | 6  17 | 20  94 | 0.341 |
| **AFP (ng/mL)**  ≤ 20  ＞20 | 19  43 | 22  53 | 0.867 | 5  16 | 36  80 | 0.506 | 9  32 | 33  72 | 0.723 | 26  60 | 14  37 | 0.729 | 8  15 | 33  81 | 0.577 |
| **Tumor size (cm)**  < 5  ≥ 5 | 13  49 | 6  69 | **0.029*** | 3  18 | 17  99 | 0.965 | 7  25 | 92  13 | **<0.001*** | 13  73 | 6  45 | 0.583 | 5  18 | 14  100 | 0.231 |
| **Tumor number**  single  multiple | 35  27 | 45  30 | 0.675 | 14  7 | 66  50 | 0.476 | 22  10 | 59  46 | 0.206 | 57  30 | 24  27 | 0.034 | 14  9 | 66  48 | 0.792 |
| **Lympho-invasion**  No  Yes | 56  6 | 69  6 | 0.730 | 18  3 | 107  9 | 0.330 | 29  3 | 96  9 | 0.888 | 80  6 | 45  6 | 0.338 | 20  3 | 105  9 | 0.426 |
| **Metastasis &**  **invasion**  No  Yes | 58  4 | 68  7 | 0.537 | 20  1 | 106  10 | 0.549 | 31  1 | 95  10 | 0.457 | 83  3 | 43  8 | **0.011*** | 20  3 | 106  8 | 0.332 |
| **PVTT**  No  Yes | 42  20 | 63  12 | **0.025*** | 14  7 | 91  25 | 0.240 | 25  7 | 80  25 | 0.821 | 64  22 | 41  10 | 0.424 | 17  6 | 88  26 | 0.735 |
| **TNM**  Ⅰ, Ⅱ  Ⅲ, Ⅳ | 17  45 | 23  52 | 0.677 | 6  15 | 34  82 | 0.945 | 11  21 | 26  79 | 0.248 | 27  59 | 13  38 | 0.462 | 11  12 | 30  84 | **0.031*** |

**Note:** **p-*value indicates statistically significant.

Abbreviation: HBsAg, hepatitis B surface antigen; AFP, alpha fetoprotein; PVTT, portal vein tumor thrombus.
